# Exercise Influence on Hippocampal Function: Possible Involvement of Orexin-A

**DOI:** 10.3389/fphys.2017.00085

**Published:** 2017-02-14

**Authors:** Sergio Chieffi, Giovanni Messina, Ines Villano, Antonietta Messina, Maria Esposito, Vincenzo Monda, Anna Valenzano, Fiorenzo Moscatelli, Teresa Esposito, Marco Carotenuto, Andrea Viggiano, Giuseppe Cibelli, Marcellino Monda

**Affiliations:** ^1^Section of Human Physiology and Unit of Dietetic and Sport Medicine, Department of Experimental Medicine, Second University of NaplesNaples, Italy; ^2^Department of Clinical and Experimental Medicine, University of FoggiaFoggia, Italy; ^3^Department of Mental Health, Physical and Preventive Medicine, Clinic of Child and Adolescent Neuropsychiatry, Center for Childhood Headache, Second University of NaplesNaples, Italy; ^4^Department of Medicine and Surgery, University of SalernoSalerno, Italy

**Keywords:** hippocampus, plasticity, neurogenesis, exercise, orexins, cognition, mood, depression

## Abstract

In the present article, we provide a brief review of current knowledge regarding the effects induced by physical exercise on hippocampus. Research involving animals and humans supports the view that physical exercise, enhancing hippocampal neurogenesis and function, improves cognition, and regulates mood. These beneficial effects depend on the contribute of more factors including the enhancement of vascularization and upregulation of growth factors. Among these, the BDNF seems to play a significant role. Another putative factor that might contribute to beneficial effects of exercise is the orexin-A. In support of this hypothesis there are the following observations: (1) orexin-A enhances hippocampal neurogenesis and function and (2) the levels of orexin-A increase with physical exercise. The beneficial effects of exercise may represent an important resource to hinder the cognitive decline associated with the aging-related hippocampal deterioration and ameliorate depressive symptoms.

## Introduction

For many years, researchers believed that neurogenesis, i.e., the production of new neurons through the division of stem cells within the brain, takes place only during embryonic development and not when the brain is fully developed. However, in recent decades, experimental evidence has shown that neurogenesis occurs also in the adult brain in two particular regions: the olfactory bulb, involved in the perception of odors, and the hippocampus, mainly involved in memory consolidation (Whitman and Greer, 2009; Kempermann et al., 2015). In the hippocampus, multipotent undifferentiated neural stem cells, located in the subgranular zone of the dentate gyrus, give rise to neural progenitor cells. These cells proliferate and migrate into the granule cell layer and differentiate into neurons, astroglia, or oligodendrocytes. The newborn neurons project into the CA3 region where they are integrated in functional circuits (Gage, 2000; Kempermann et al., 2004). A seminal study by Eriksson et al. (1998) provided direct evidence for adult neurogenesis in humans. These authors (Eriksson et al., 1998) obtained postmortem human brain tissue from adult patients who received, for diagnostic purposes, bromodeoxyuridine that labels DNA during the S phase. Eriksson et al. (1998) demonstrated that new neurons were generated from dividing progenitor cells in the dentate gyrus. Spalding et al. (2013) estimated that about 700 new neurons are added in each hippocampus per day.

## Exercise and hippocampus: animal model

Hippocampal neurogenesis is favored by many factors, including environmental enrichment and voluntary exercise and associative learning (Kempermann et al., 1997, 1998; van Praag et al., 1999a,b, 2005). Regarding the enriched environment, it is a complex combination of social, cognitive, and physical stimulations. van Praag et al. (1999b) attempted to disentangle such components and observed that voluntary exercise doubled the number of surviving newborn cells in amounts similar to enrichment condition. Then, van Praag et al. (1999b) suggested that voluntary exercise was sufficient for enhanced neurogenesis in the adult mouse dentate gyrus. Interestingly, the effect of exercise on neurogenesis appears to be restricted to the hippocampus. This was demonstrated by Brown et al. (2003) who found that voluntary exercise selectively doubled the amount of new granule cells in the hippocampus, but it did not modify the number of adult-generated olfactory granule cells. Another effect of exercise is the increase of cerebral blood volume of the dentate gyrus in mice (Pereira et al., 2007). This increase was considered an *in vivo* correlate of neurogenesis since it correlated with postmortem measurements of neurogenesis (Pereira et al., 2007). Hippocampal neurogenesis diminishes with aging (Kuhn et al., 1996; Heine et al., 2004). However, van Praag et al. (2005) suggested that this decrease may be partially opposed by exercise. The Authors (van Praag et al., 2005) reported that in aged running mice voluntary exercise enhanced hippocampal neurogenesis and learning. Interestingly, the morphology of new neurons did not differ between young and aged runners, supporting the hypothesis that local hippocampal environment of the aged dentate gyrus is effective in sustaining neurogenesis (van Praag et al., 2005). Some researchers investigated the effects of hyppocampal lesion on behavioral performance. Clark et al. (2008) irradiated with gamma rays the region of mice hippocampus reducing neurogenesis by 50%, whereas in non-irradiated animals running increased neurogenesis fourfold. Furthermore, irradiation selectively eliminated gains in water maze performance that depends on hippocampus. However, the decrease in neurogenesis and cognitive skills induced by irradiation could be mitigated by exercise (Ji et al., 2014). Rats who received whole-brain irradiation and, following irradiation, were forced to perform exercise showed a significant amelioration of the impaired neurogenesis and cognition (Ji et al., 2014).

The morphological and functional changes in hippocampus produced by exercise likely depend on the contribute of different factors, including the enhancement of vascularisation, the involvement of growth factors and the regulation of the expression in a variety of gene transcripts. Exercise upregulates expression of brain-derived neurotrophic factor (BDNF), vascular endothelial growth factor (VEGF), and insulin-like growth factor-1 (IGF-1). Among these, the BDNF is considered to be the most important factor. A lot of studies suggest that the upregulation of BDNF play an significant role in hippocampal neurogenesis, synaptic plasticity and learning (Neeper et al., 1995; Cotman and Berchtold, 2002; Vaynman et al., 2004; Cotman et al., 2007).

## Exercise and hippocampus: humans

An interesting and fruitful line of research in recent years has investigated the influence of exercise on cognitive functions in humans. Exercise may enhance cognitive functions both in young, e.g., improving verbal memory and performance in a map recognition (Grego et al., 2005; Pereira et al., 2007; Winter et al., 2007), and in older adults, e.g., enhancing efficiency of attentional (Kramer et al., 1999) and executive-control processes (Colcombe and Kramer, 2003). Pereira et al. (2007) found that in humans (21–45 years) exercise selectively increased the cerebral blood volume of the dentate gyrus. This increase correlated with improved verbal memory. Intriguingly, Griffin et al. (2011) found that acute and chronic exercise enhanced the performance of young (22 ± 2 years), sedentary (i.e., not involved in any regular physical training) men in the face–name matching task (associative memory) and not in the Stroop task (executive functions). Note that face–name matching task recruits the hippocampus and associated structures of the medial temporal lobe (Zeineh et al., 2003; Kirwan and Stark, 2004), whereas the Stroop word–color task the anterior cingulate cortex and other frontal regions (Leung et al., 2000). However, other researchers reported post-exercise improvements also in the performance of the Stroop word and color tests (Ferris et al., 2007).

As concerns cognitive functions in older adults, in general, healthy older adults with higher fitness levels have less cognitive decline (Yaffe et al., 2001; Barnes et al., 2003) and reduced risk for dementia and Alzheimer's disease (Podewils et al., 2005; Larson et al., 2006) than those with lower fitness levels. In older humans, imaging studies showed that exercise not only spared brain volume but also increased both gray and white matter volume in the prefrontal and temporal cortices, i.e., those same regions that are often reported to deteriorate with aging (Colcombe et al., 2006; Rosano et al., 2010) and be severely affected in Alzheimer's disease (Galeone et al., 2011; Chieffi et al., 2014a,b, 2015; Chieffi, 2016a). The integrity of these regions play central roles in successful everyday functioning. Prefrontal regions are associated with working memory and executive functions (Chieffi et al., 2008, 2012; Godefroy et al., 2008; Roca et al., 2010) and temporal lobes with long-term memory function (Jeneson and Squire, 2011; Lech and Suchan, 2013).

Exercise has also beneficial effects on the hippocampus, a brain region particularly sensitive to age-related decay. Hippocampus shrinks with age (Raz et al., 2005) and its atrophy predicts shorter time-to-progression from mild cognitive impairment to Alzheimer's dementia (Jack et al., 2010). Erickson et al. (2011) reported that older (55–80 years) individuals with higher levels of aerobic fitness were associated with greater volume of the hippocampus and displayed better spatial memory performance than individuals with lower fitness levels. One year aerobic exercise intervention was effective in increasing the size of the anterior hippocampus by 2% (Erickson et al., 2011), contrasting with the reported 1–2% annual hippocampal volume shrinkage in older adults without dementia (Raz et al., 2005). Gains in hippocampal blood flow and memory performance were also observed in healthy sedentary adults (57–75 years) with shorter term exercise (3 months) by Chapman et al. (2013). More researches reported that exercise increased BDNF concentrations in the serum suggesting a key role for this neurotrophic factor in enhancing hippocampal volume and cognitive function (Ferris et al., 2007; Erickson et al., 2011; Griffin et al., 2011).

## Exercise, orexin, and hippocampus

Another factor that acting on the hippocampus might contribute to the beneficial effects of physical exercise on cognition is the orexin-A. The orexin-A/hypocretin-1 (OxA/Hcrt-1) and orexin-B/hypocretin-2 (OxB/Hcrt-2) are neuropeptides synthesized by a cluster of neurons in the lateral hypothalamus. Orexins selectively act on two G protein-coupled receptors: the orexin/hypocretin 1 receptor (Ox1R/HcrtR1), which has higher affinity to orexin-A, and the orexin/hypocretin 2 receptor (Ox2R/HcrtR2), which has equal affinity to both orexin-A and orexin-B (Sakurai et al., 1998; Scammell and Winrow, 2011). Ox1R and Ox2R are generally excitatory, being the common cellular response to their activation an increase of intracellular calcium (Gotter et al., 2012). Furthermore, orexin receptors may mediate both acute and long-lasting effects (Scammell and Winrow, 2011). Acute effects depend on several ionic mechanisms, such as the inhibition of potassium channels and the activation of a sodium/calcium exchanger; long-lasting effects by increasing the number of *N*-methyl-*D*-aspartate (NMDA) receptors in the cell membrane and making the neurons more responsive to the excitatory effects of glutamate for several hours (Scammell and Winrow, 2011). Ox1Rs are widely expressed throughout the brain, including hippocampal formation, dorsal raphe nucleus and locus coeruleus, while Ox2Rs are found mainly in the cerebral cortex, nucleus accumbens, and subthalamic and paraventricular thalamic nuclei (Trivedi et al., 1998; Marcus et al., 2001). Orexinergic neurons receive a variety of signals related to environmental, physiological, and emotional stimuli (Yoshida et al., 2006; Scammell and Winrow, 2011; Marra et al., 2013; Franco et al., 2014), and project broadly to the entire CNS. Orexinergic projections are involved in regulating wakefulness and arousal (Saper et al., 2005), motivation and emotions (Sakurai and Mieda, 2011; Thompson and Borgland, 2011; Boscia et al., 2015), and motor and autonomic functions (Nattie and Li, 2012; Messina et al., 2013, 2014b, 2015a,b; Messina A. et al., 2016). Orexinergic system may also induce structural changes in the hippocampus influencing hippocampal learning and memory processes. In support of this view Wayner et al. (2004) reported that local dentate gyrus perfusion with orexin-A enhanced long-term potentiation (LTP) in anesthetized rats, suggesting that orexins positively regulate hippocampal synaptic plasticity. Furthermore, the authors (Wayner et al., 2004) showed that this improvement was blocked when rats were pretreated with SB-334867, a specific Ox1R antagonist (Wayner et al., 2004). The effects of dentate gyrus-OX1Rs antagonization on LTP occurred also in freely moving rats (Akbari et al., 2011). Subsequently, Akbari et al. (2007, 2008) showed that blockade of Ox1Rs with SB-334867 in CA1 and in dentate gyrus regions impaired spatial memory in Morris water maze, suggesting endogenous orexin-A positively modulated the performance of learning tasks via Ox1Rs. Some studies examined the effects of orexin-A in rats in which the administration of Pentylenetetrazol (PTZ) induced seizures resulting in the hippocampal atrophy, learning and memory deficits and decrease of cerebrospinal fluid-level of orexin-A. Note that the levels of orexin-A in cerebrospinal fluid are decreased in patients after repetitive seizures (Rejdak et al., 2009). Zhao et al. (2014) observed that the intracerebroventricular (i.c.v.) injection of orexin-A in PTZ-kindled rats attenuated the impairment of spatial learning and memory. Furthermore, they (Zhao et al., 2014) showed that orexin-A enhanced neurogenesis in the dentate gyrus promoting neuronal proliferation and differentiation. Interestingly, in rats treated with orexin-A more than 50% of newborn cells differentiated into neurons, but only ~30% of the newborn cells differentiated into neurons in the control group. This suggested that orexin-A not only stimulated cell proliferation but also promoted the differentiation of newborn cells (Zhao et al., 2014). Recently, Yang et al. (2013) showed that orexin-A is also implicated in social memory, i.e., the ability to distinguish and remember familiar from novel conspecifics. The authors (Yang et al., 2013) used orexin/ataxin-3-transgenic (AT) mice, in which orexin neurons degenerate by 3 months of age (Hara et al., 2001). Compared with their wild-type (WT) littermates, AT mice displayed deficits in long-term social memory. However, nasal administration of exogenous orexin-A restored social memory and enhanced synaptic plasticity in the hippocampus (Yang et al., 2013). Interestingly, Yang et al. (2013) found in the AT hippocampus an attenuation of LTP and a decrease of phosphorylated CREB (pCREB) levels. The authors (Yang et al., 2013) suggested that the alteration of these processes might underlie the long-term social memory deficit in AT mice. According previous studies showed that the formation of long-term memory in several hippocampus-dependent cognitive tasks involve CREB phosphorylation (Kogan et al., 2000; Nomoto et al., 2012) and neurotransmitters such as dopamine, serotonin, and acetylcholine, which enhance memory, induce CREB phosphorylation (Kogan et al., 2000; Shirayama and Chaki, 2006). Recently, it was reported that also orexin-B can improve memory processes. Palotai et al. (2014) showed that the i.c.v. administration of orexin-B in rats improved learning, memory consolidation and retrieval, in a dose-dependent manner. The action of orexin-B on memory functions was further supported by the observation that rats pretreated with the EMPA (N-ethyl-2-[(6-methoxy-pyridin-3-yl)-(toluene-2-sulphonyl)-amino]-N-pyri-din-3-ylmethyl-acetamide), a selective Ox2R antagonist, reversed completely memory consolidation.

Physical exercise produces an increase of orexin-A level in cerebrospinal fluid of rats (Martins et al., 2004), dogs (Wu et al., 2002), and cats (Kiyashchenko et al., 2002). An increase of plasmatic orexin-A with exercise was reported in humans (Messina et al., 2014a; Messina G. et al., 2016). Messina et al. (2014a) collected blood samples from adult participants before (time 0 min) and after (times 15 and 30 min) a cycle ergo meter exercise at 75 W for 15 min. Results showed that the post-exercise values of orexin-A were significantly higher compared to pre-exercise values. The source of peripheral orexin is still unclear. Tsunematsu and Yamanaka (2012) proposed that orexin A might be directly released from the pituitary into the blood stream, since orexin-immunoreactive fibers are present in the median eminence and pituitary (Date et al., 1999), or leaked from the cerebrospinal fluid. Furthermore, orexin-A may rapidly cross the blood-brain barrier from brain tissue to reach blood by the process of simple diffusion, being highly lipophilic (Kastin and Akerstrom, 1999). Peripheral tissues may represent another source of peripheral orexin. Orexin-immunoreactive cells are observed in the gastrointestinal tract and pancreas (Nakabayashi et al., 2003).

As a whole the experimental data we have reported allow to hypothesize that the increase of orexin-A levels with exercise may contribute to improve cognition, enhancing hippocampal plasticity, and function.

Experimental evidence also suggests that physical exercise, besides improving cognition, has beneficial effects on mood regulation. In support of an antidepressant effect of exercise there are exhaustive reviews (Blake et al., 2009; Bridle et al., 2012; Mura et al., 2014; Kvam et al., 2016). Interestingly, patients with depression showed smaller hippocampal volumes (Steffens et al., 2000; Sheline, 2003) and an increase in hippocampus volume following exercise was positively associated with depressive symptoms improvement (Krogh et al., 2014).

Orexin-A, as well as BDNF, might contribute to beneficial effects on mood regulation induced by exercise (Chieffi, 2016b). Wistar-Kyoto (WKY) rats represent a genetic animal model of depression. They have fewer (about 18%) and smaller (about 15%) orexin-A neurons in the hypothalamus compared to control Wistar rats (Allard et al., 2004). These observations were in line with the observation of Taheri et al. (2001) who reported a decrease of about 22% in hypothalamic prepro-orexin mRNA in WKY rats. Some studies have investigated the links between orexins, depression, and hippocampal neurogenesis. Arendt et al. (2013) found that mice displaying an increase of immobility in the forced swim test (FST), a commonly used measure of depressive behavior, had lower hippocampal expression of orexin-A. Furthermore, the i.c.v. administration of orexin-A led to a significant reduction in animal immobility in the FST and an increase in the number of cells in the dentate gyrus (Ito et al., 2008). Ito et al. (2008) suggested that the enhancement of cell proliferation in the dentate gyrus by orexin-A might have an antidepressive-like effect. Furthermore, the treatment with the OXR1 antagonist SB-334867 blocked both the orexin-A-induced decrease in the FST immobility and the increase in the number of cells in the dentate gyrus. In humans, Brundin et al. (2007a) showed that suicidal patients with major depression exhibit significantly lower orexin-A levels in the cerebrospinal fluid. In addition, low levels of orexin-A in the cerebrospinal fluid are related to pronounced symptoms of inertia and lassitude in suicide attempters (Brundin et al., 2007b).

Experimental evidence suggests the BDNF may have antidepressant-like effects. Shirayama et al. (2002) showed that the infusion of BDNF into the rat hippocampus decreased immobility in the FST. Furthermore, Karege et al. (2005) found that suicidal patients with depression had reduced BDNF levels in their hippocampus. An important question is whether the orexin and BDNF mechanisms interact. To our knowledge, this issue has been addressed only by Yamada et al. (2009) who applied orexin-A and orexin-B to cortical neuron cultures. They found that orexin-B, but not orexin-A, increased the mRNA expression of BDNF.

**Figure 1:**
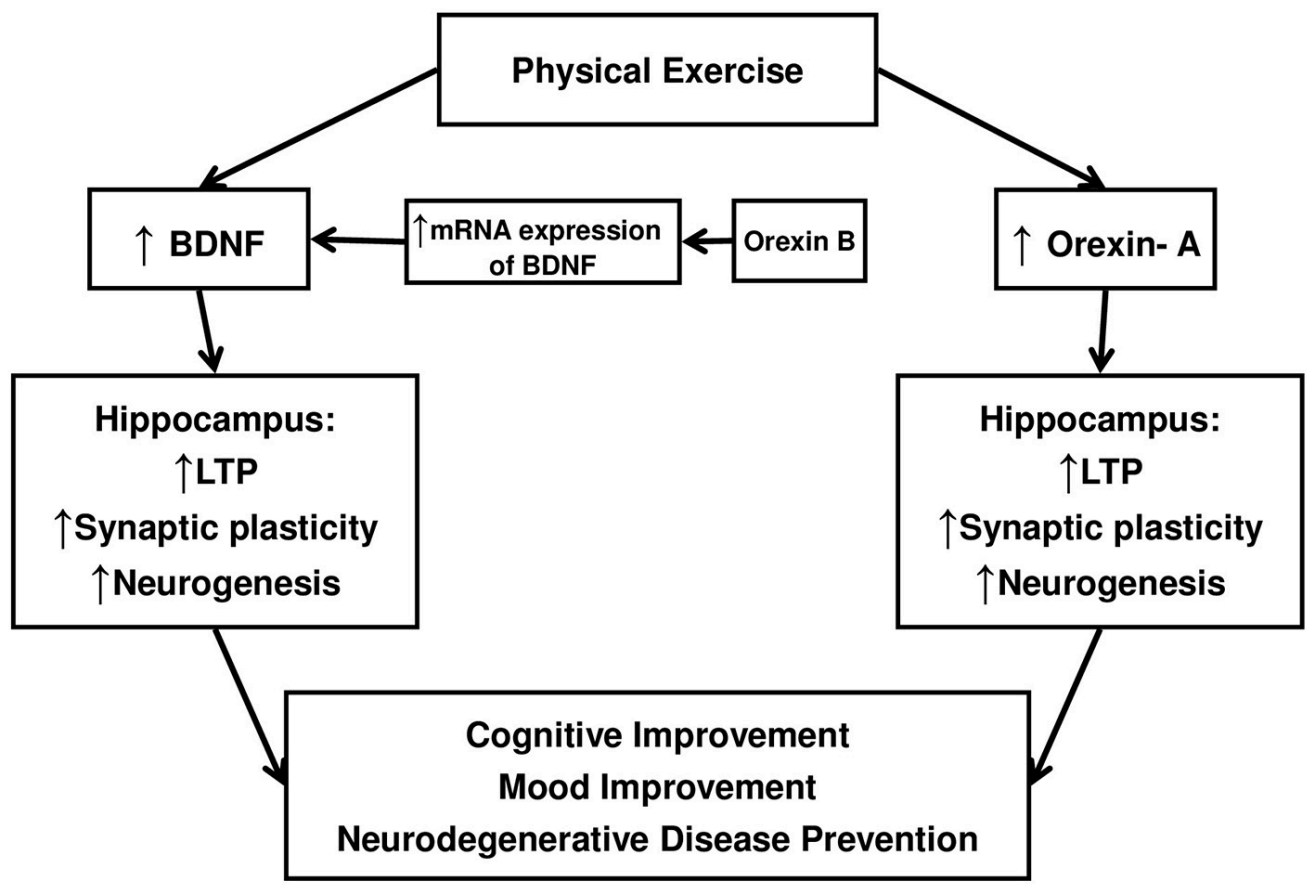
**A Schematic diagram to illustrate as Orexin-A and BDNF might contribute to cognitive and mood improvements induced by exercise**.

Taken together, the experimental observations we reported support the view that the orexin-A, as well as the BDNF, might contribute to the beneficial effects of exercise on mood regulation (see Figure 1).

## Conclusions

In this brief review, we have reported studies that support the view that physical exercise is an effective tool for enhancing cognitive performance and regulating mood. Exercise would induce morphological and functional changes of brain regions that play central roles in successful everyday functioning, such as frontal and temporal cortices, and the hippocampus located in the inner (medial) region of the temporal lobe. The frontal lobe is critical for executive functions (Chieffi et al., 2004, 2009; Iavarone et al., 2007), the temporal lobe for long-term memory skills (Jeneson and Squire, 2011; Lech and Suchan, 2013). The study of exercise-induced hippocampal changes has particularly attracted the interest of many research groups as the hippocampus, along with the olfactory bulb, is the place in the adult in which mammalian brain continues to generate new neurons throughout life (Whitman and Greer, 2009; Kempermann et al., 2015). Thus, it is very important to define accurately the factors that support neurogenesis and are enhanced by exercise. The factors most likely involved in exercise-induced hippocampal changes are the microcirculation and the production of growth factors. Another putative factor that might contribute to the beneficial effects of exercise is the orexin-A. In favor of this hypothesis, as previously reported, there are the following observations: (1) orexinergic neurons have connections to regions involved in cognition and mood regulation such as the hippocampus; (2) orexin-A enhances hippocampal neurogenesis and functions; (3) orexin-A levels increase with exercise. However, currently several important questions remain unanswered: Is the orexin-A necessary for hippocampal neurogenesis? Does the systemic administration of orexin-A mimic exercise-induced effects related to neurogenesis, hippocampal structure and function? Does the orexin-A mechanism relate to other mechanisms? E.g., as suggested by an anonymous reviewer, does the administration of an orexin-A antagonist also inhibit exercise-induced increases in BDNF? Future experiments are needed to answer these questions.

Interestingly, regions that benefit from exercise are also those same regions that deteriorate with aging, loading to a decline in a broad array of cognitive processes. Several studies found that exercise is an effective tool in slowing cognitive decline (Erickson et al., 2011; Chapman et al., 2013) and in emotional regulation (Blake et al., 2009; Mura et al., 2014; Kvam et al., 2016). Given the projected increase in the number of adults surviving to advanced age, and the staggering costs of caring for older individuals who suffer from neurological decline and mood disorders, physical activity may represent a simple, but effective and low cost, therapeutic intervention to improve neurocognitive and emotional functions. Furthermore, physical activity is accessible to most adults and is not plagued by intolerable side effects often found with pharmaceutical treatments.

## Author contributions

SC, GM, MC, and AV carried out the study; IV, AM, ME, VM, AV, FM, and TE participated in the design of the study; SC, GM, AVi, GC, and MM participated in its design and coordination and helped to draft the manuscript. All authors read and approved the final manuscript.

### Conflict of interest statement

The authors declare that the research was conducted in the absence of any commercial or financial relationships that could be construed as a potential conflict of interest.
